# Primary unilateral cleft lip repair

**DOI:** 10.4103/0970-0358.57189

**Published:** 2009-10

**Authors:** H. S. Adenwalla, P. V. Narayanan

**Affiliations:** Department of Plastic Surgery, Burns, Charles Pinto Centre for Cleft Lip, Palate and Craniofacial Anomalies, Jubilee Mission Medical College & Research Institute, Trichur-680 005, Kerala, India

**Keywords:** Closed alar dissection, Notch-free vermillion, Primary septoplasty, Unilateral cleft lip

## Abstract

The unilateral cleft lip is a complex deformity. Surgical correction has evolved from a straight repair through triangular and quadrilateral repairs to the Rotation Advancement Technique of Millard. The latter is the technique followed at our centre for all unilateral cleft lip patients. We operate on these at five to six months of age, do not use pre-surgical orthodontics, and follow a protocol to produce a notch-free vermillion. This is easy to follow even for trainees. We also perform closed alar dissection and extensive primary septoplasty in all these patients. This has improved the overall result and has no long-term deleterious effect on the growth of the nose or of the maxilla. Other refinements have been used for prevention of a high-riding nostril, and correction of the vestibular web.

The unilateral cleft lip in its varying manifestations of shape, size and asymmetry is a complex deformity. To obtain consistent results one requires basic training in soft tissue handling, an understanding of the bony foundations of the face, followed by experience and a fair amount of craftsmanship.

In the late 1950s the senior author was introduced by his mentor Charles Pinto to the straight repair of Rose[1] and Thompson[2] as modified by Peet,[3] who called it the “Oxford modification of the straight repair”. In the hands of the artistic Peet it gave good results.

In his search for something better, Charles Pinto brought back from Barrett Brown's unit at St Louis, Missouri, a form of the triangular flap of Mirault[4] that had been modified by Vilray Papin Blair, Brown and Mc Dowell[5,6] into a smaller triangular flap. The Blair–Brown–McDowell plan held centre stage for a good 10 years. The stature of these three great men and their artistry was probably one of the reasons why this procedure flourished. In our hands the results were no better than the straight repair. There was not the slightest semblance of a Cupid's bow in these repairs; instead there was an unnatural central peak and in most cases a tight lip resulted. Secondary corrections of these lips were always difficult.

A major breakthrough in cleft surgery took place when Le Mesurier,[7] an orthopaedic surgeon working at the Hospital for Sick Children at Toronto, used Werner Hagedorn's quadrilateral flap[8,9] and for the first time created a Cupid's bow. No surgeon at the time could ignore the positive advantages of having a nice Cupid's bow. As time went on and the long-term results of the Le Mesurier repair were shown at conferences, it became obvious that the lip on the cleft side became long and over-hanging and the scar, like the triangular flap was unnatural and did cut across the normal philtral line.

As the ‘Le Mesurier’ began to fade out, Tennison's modification[10] with a Z plasty began to be accepted. Peter Randall[11] did to the Tennison what Blair and Brown had done to the Mirault - he made his triangle smaller and marked his points with greater precision. Sawhney[12] of Chandigarh improved on the Tennison-Randall's operation, making the cutting of the triangular flap almost geometrical in its precision. With Sawhney's contribution, the triangular flap became easy to teach and easy to execute and is still quite popular with surgeons in North India. When well executed, the Tennison-Randall-Sawhney procedure gives good results. The scar however is unacceptable and, when not properly executed, secondary repairs are difficult.

Somehow, we at the Charles Pinto Centre, missed out on the Tennison-Randall-Sawhney improvements and went straight on to the rotational advancement technique of Millard. In 1958, on his last visit to India, Sir Harold Gillies demonstrated the rotational advancement technique to a group of Indian surgeons at Pune. He turned around to the fascinated audience and said “Gentlemen, try this one - I think it has merit, but I must warn you that it has not yet been published!” The Millard procedure[13,14] broke like dawn on the Indian horizon and caught the imagination of surgeons the world over by its clear, logical thought process. Millard said that:

- All the previous flap procedures based their logic on the false premise that the actual defect in the cleft is in the lower third of the lip, which is not so. Discarding precious tissue in Tennison's approach when there was already poverty, is against all established plastic surgical principles.
- Three quarters of the Cupid's bow is present on the non-cleft side, but is riding high. What better way of bringing it down in a horizontal line with its fellow than by a rotational flap? No rotational flap is complete without a back cut and this not only further helps to drop the obliquely oriented Cupid's bow, but compensates for the contracture of the straight line of the Millard procedure.
- This main rotational flap is taken from the rich non-cleft side and not from the poverty stricken cleft side as in the triangular and quadrilateral flap procedures. (“It is unwise to borrow from Peter to pay Paul when Peter can ill afford it”).[15]
- The defect thus created is in the upper part of the lip and can be hidden under the overhanging nostril.
- What better way of filling this defect than by advancing a flap from the cleft side.
- The advancement flap gives the additional bonus of correcting the nostril flare.
- The “C” flap helps to lengthen the short columella.
- The scar imitates the philtral line, creates a philtral column, a philtral dimple and a slight pout which adds charm to the finished result. The scars of both the triangular and quadrilateral flaps crisscross Langer's lines, which again is contrary to basic tenets of plastic surgery.

This to our mind is the eight-fold path to the ‘Cleft Nirvana” that the reconstructive surgeon wants to achieve.

The authors would not like to give an impression that mere reading of these eight points would ensure a good result. The Millard procedure needs to be taught on the table, needs a considerable amount of virtuosity on the part of the surgeon and it needs a fair amount of experience. Unlike the ‘Tennison–Sawhney’ there are very few mathematically precise points to mark and you can “cut as you go” depending upon the needs of the case, keeping your eye on shape and symmetry. As Millard remarked “all art depends on freedom for its vitality for no two lips are identical - they may be similar but never identical”.[14] The straight line part of the Millard incision often contracts and pulls the Cupid's bow up in the first few months, but in a year's time it descends without any further intervention. [Figure 1a–c]

**Figure 1a:**
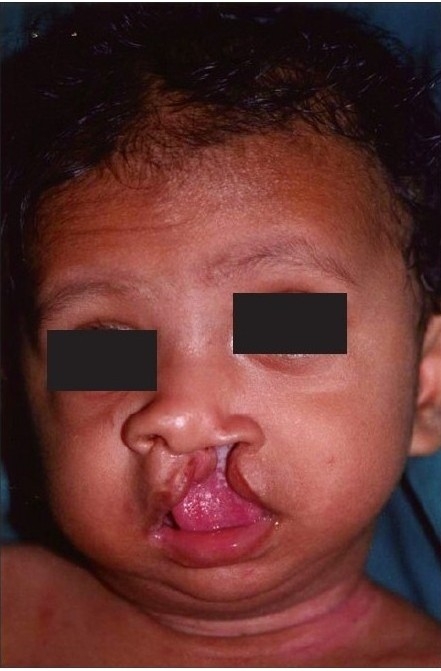
Pre-operative

**Figure 1b:**
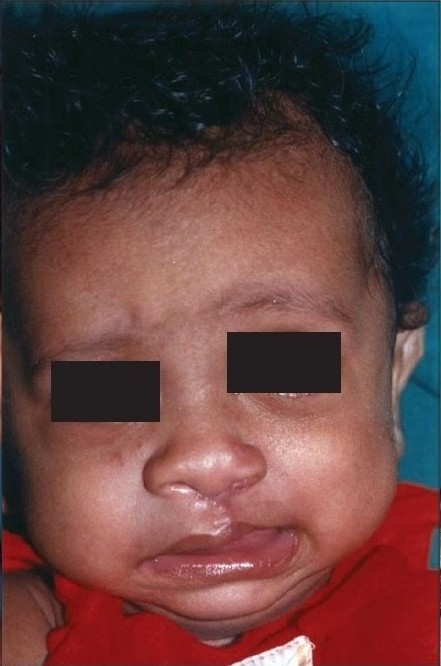
Early post operative

**Figure 1c:**
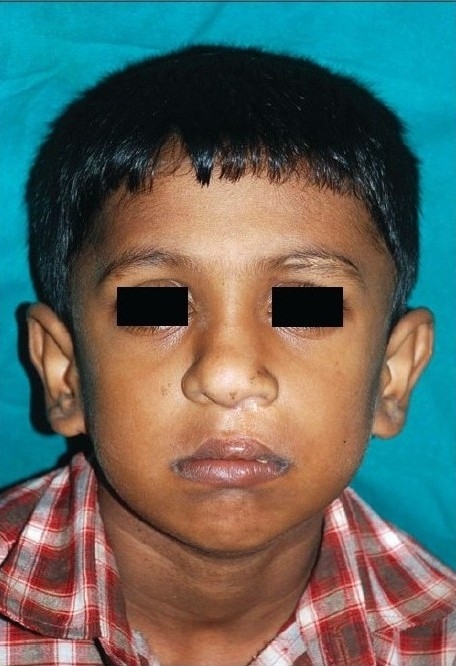
Delayed post operative

Critics of the Millard operation have often said in publications that the rotational advancement procedure is only suitable for partial clefts and not for complete ones.[14] This statement is far from the truth. To the original Millard theorem, in this presentation, we have added our own original method of correcting the nostril deformity and our method of avoiding a notch or whistle deformity on the vermillion.

## PROTOCOL

### Timing of Surgery

The “rule of tens”[16] has been followed widely in many parts of the world. However, this does not apply to our country. We are physically a smaller people and many of our children are undernourished. We undertake surgery for these children when they are at least 5 Kg in weight. On an average, our children attain this weight by five to six months. Neonatal surgery is not recommended in view of the risks involved and the need for a compromise on the surgical procedure to minimise the time and extent of the surgery. Miniature tissues are difficult to work on and work in the nose is well nigh impossible.

### Pre-surgical orthodontics

This is being followed in many centres across the world.[17–20] We believe that without any pre-surgical intervention, we are able to achieve results at least on par with those from centres using some form of pre-surgical orthodontics in unilateral cleft lips. Expense and patient compliance are also factors to be taken into account. Hence we do not use any orthodontic intervention prior to surgery.

### Procedure

**Figure 2:**
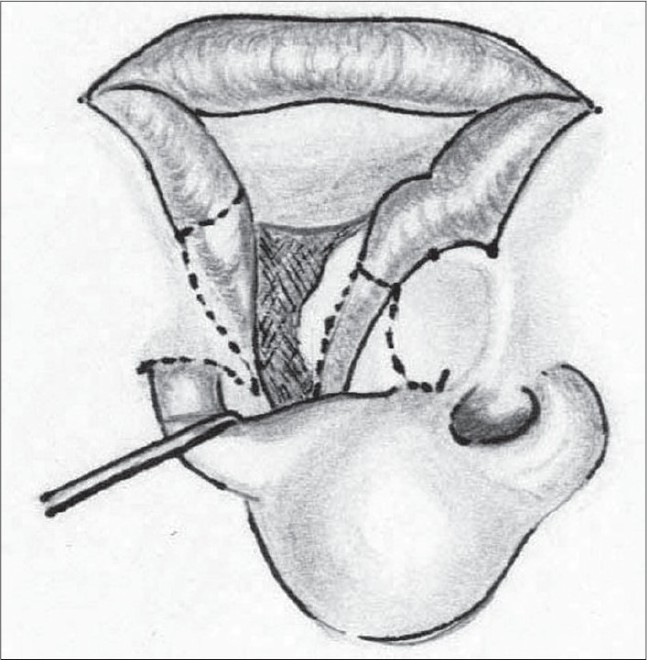
The Millard incisions marked

We use the standard Millard incisions[14] [Figure 2]. The rotation flap at its superior end hugs the base of the columella. We always make an ample back cut, taking care not to encroach onto the philtral column on the non-cleft side. An adequate rotation incision with a back-cut is required to get the Cupid's bow points at the same horizontal level. If the back cut were to transgress the philtral column on the non-cleft side this would cause a lengthening of the lip on that side.

We do use the peri-alar component of the Millard incision for the advancement flap. Many contemporary authors[21] have abandoned this, as they are apprehensive about the visibility of the scar. We have, however, used this in more than 7,000 cleft lips and we are entirely satisfied that the scar is not obvious if the incisions are placed precisely at the base of the ala in the alar groove [Figures 3a,b, 14 a,b,c, 15a,b]. The advantage of the peri-alar incision is that one can dissect the paranasal muscles under vision and include them in Millard's Cinch suture. This suture traverses the membraneous septum and takes a bite on the paranasal muscles before going back through the septum. This helps in correcting the alar flare. However, one should be careful when tightening this suture, as one can easily cause extreme narrowing and deformity of the nostril base by excessive tightening. In addition to the Millard Cinch suture, we use an additional Cinch suture with 5.0 prolene at the nasal sill. This goes through the subcutis medially, and laterally through the dermis. When this is tightened, the shape of the nostril improves significantly.

**Figure 3a:**
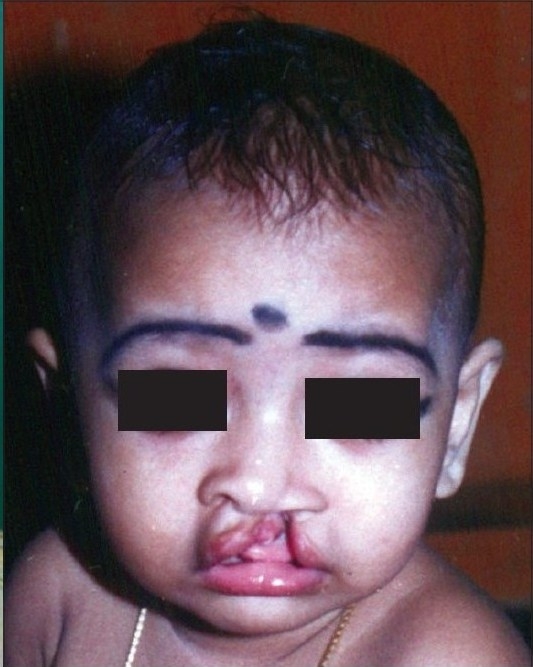
Pre-operative

**Figure 3b:**
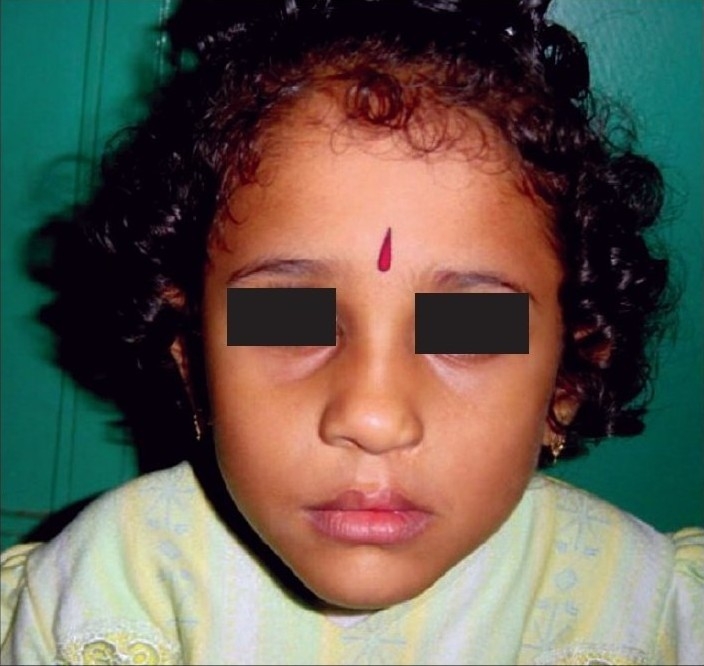
Long term post-operative

**Figure 15a:**
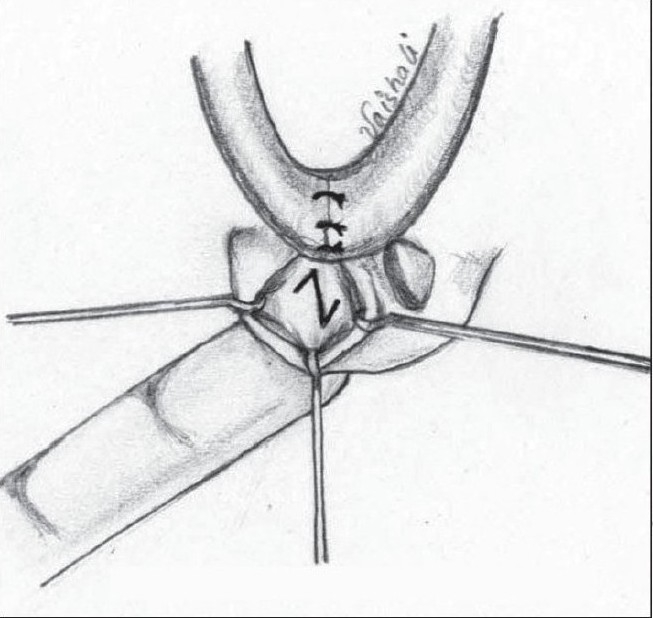
Marking of Pinto's Z plasty for the vestibular web

**Figure 15b:**
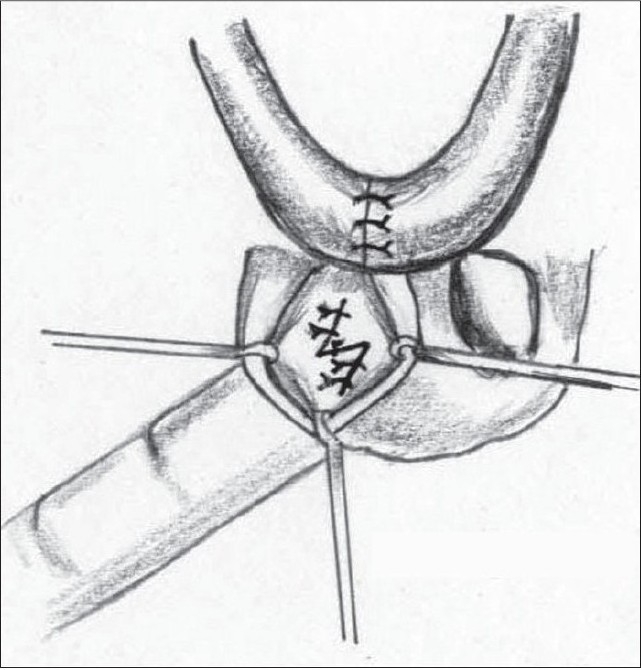
Completed Z plasty

While we basically follow the Millard technique, the senior author has included several technical refinements to the procedure. These have been in relation to the primary correction of the nasal deformity and in producing a notch-free vermillion.

### Notch-free vermillion

A notch is a common blemish following cleft lip repair. The senior surgeon at our centre analysed the main causes leading to the formation of a notch and addressed them using a protocol which has been adhered to on all unilateral cleft lip patients operated on at our centre.[22] As a result, we have been able to consistently obtain a notch-free vermillion.

### Causes of vermillion notching

- Inadequate rotation of the medial element of the lip resulting in a tented-up Cupid's bow point on the cleft side and a notch on the vermillion
- Turning-in of the sutured edges around the vermillion
- Deficiency of bulk of the orbicularis oris at the vermillion
- Contracture of the straight line scar on the mucosal aspect of the lip

Having noted the above causes, an attempt was made to correct each of them.

**Figure 4:**
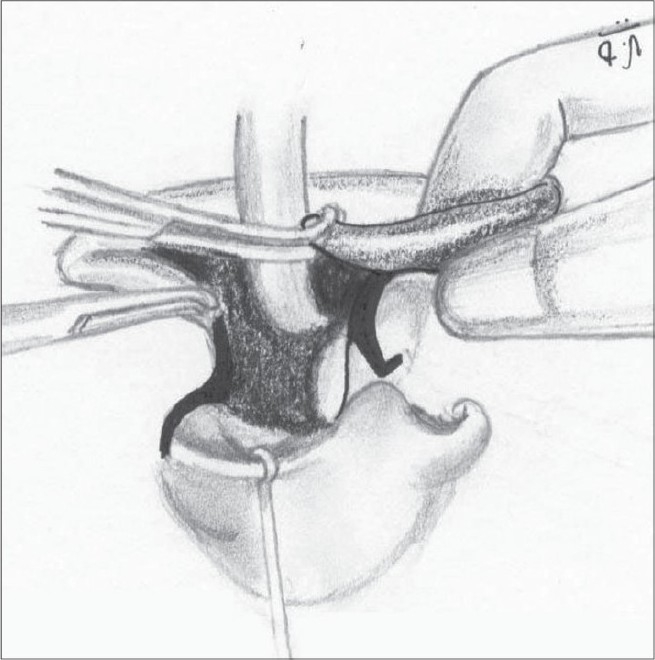
Back-cut completed. Cupid's bow peak points are at same horizontal level

**Figure 5:**
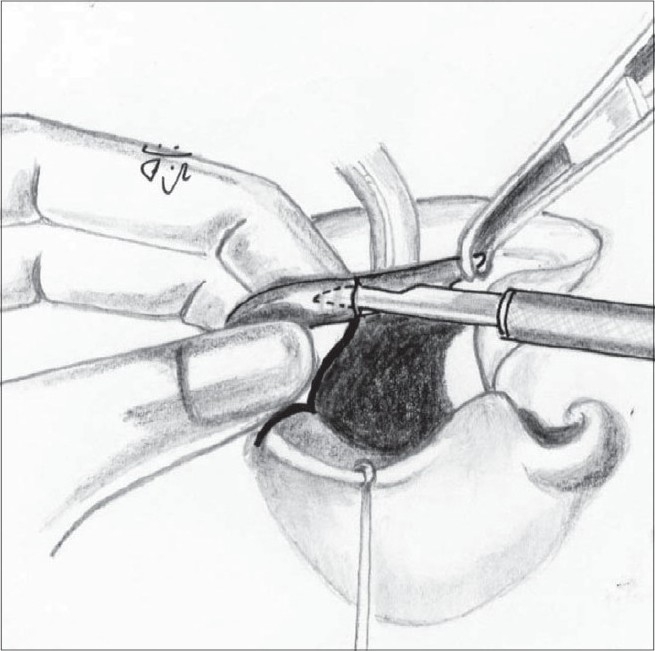
Undermining the vermillion on the cleft side. The non-cleft side vermillion is also similarly undermined

**Figure 6:**
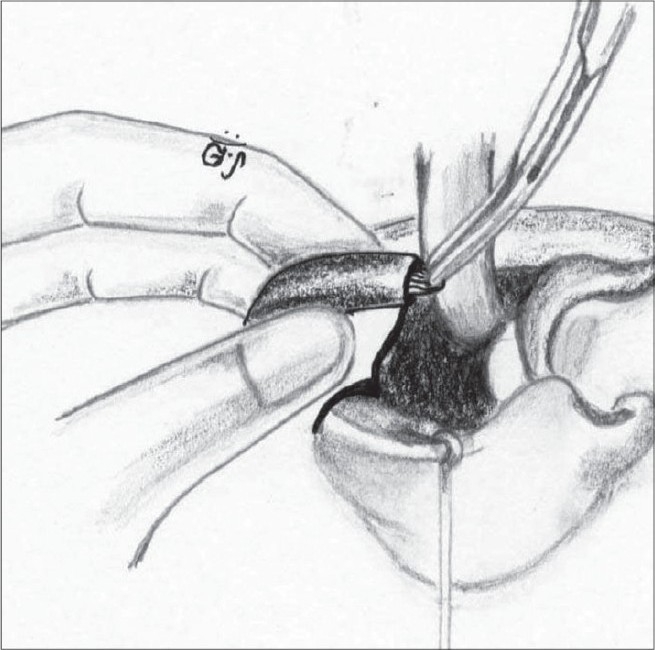
Shows the excess of orbicularis oris left behind while paring the vermillion

**Figure 7:**
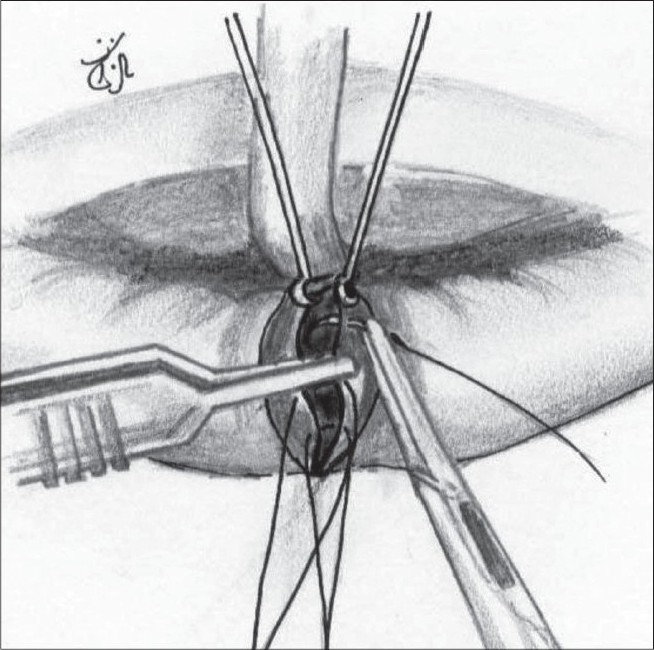
The muscle left behind on both the sides being sutured with 6.0 Nylon. A minimum of 3 stitches

**Figure 8:**
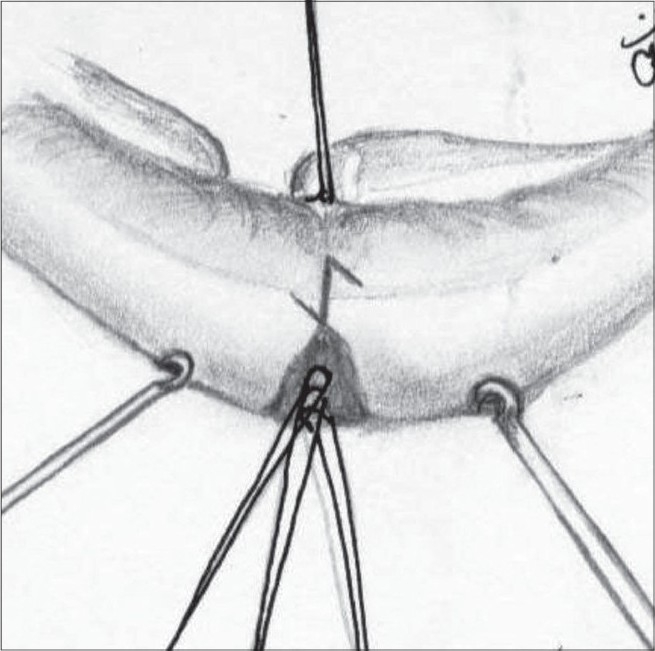
Z plasty on the mucosa

- Adequate rotation of the medial element with an ample back-cut in all patients [Figure 4].
- Undermining the skin and mucosal edges to prevent their turning in. This undermining is limited to a few millimetres from the cleft edges [Figure 5].
- While paring the vermillion, an excess of muscle tissue is retained on both the medial and lateral elements. As a result, there is a good bulk of muscle tissue that acts as a filler [Figure 6]. At least three 6/0 Nylon (Ethilon®) sutures are placed to bring this muscle together, thus creating the appearance of “a roll” or “sausage” [Figure 7].
- To counteract the straight line scar contracture, a Z plasty is mounted on the mucosal aspect of the lip and an attempt is made to align Noordhoff's red line [Figure 8].

All the above mentioned steps are done in all our unilateral cleft lip patients. The long-term results of patients operated in this manner are shown in Figures 3a,b, 1a,b,c 9a,b.

**Figure 9a:**
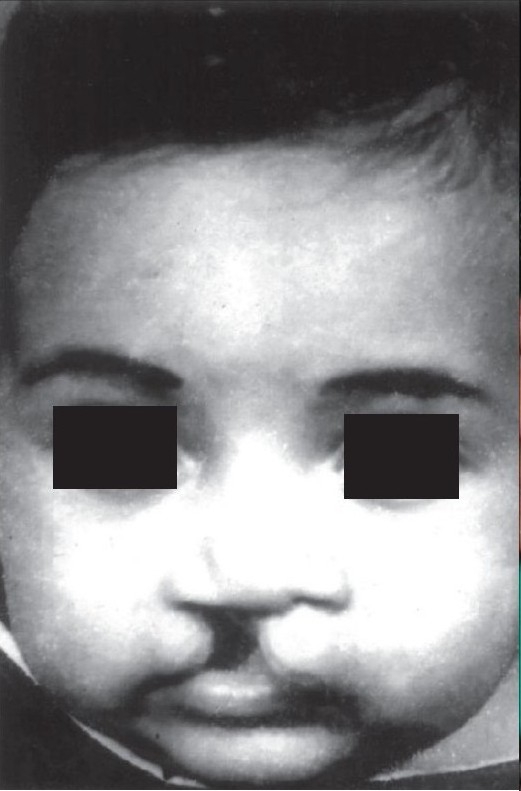
Pre-operative

**Figure 9b:**
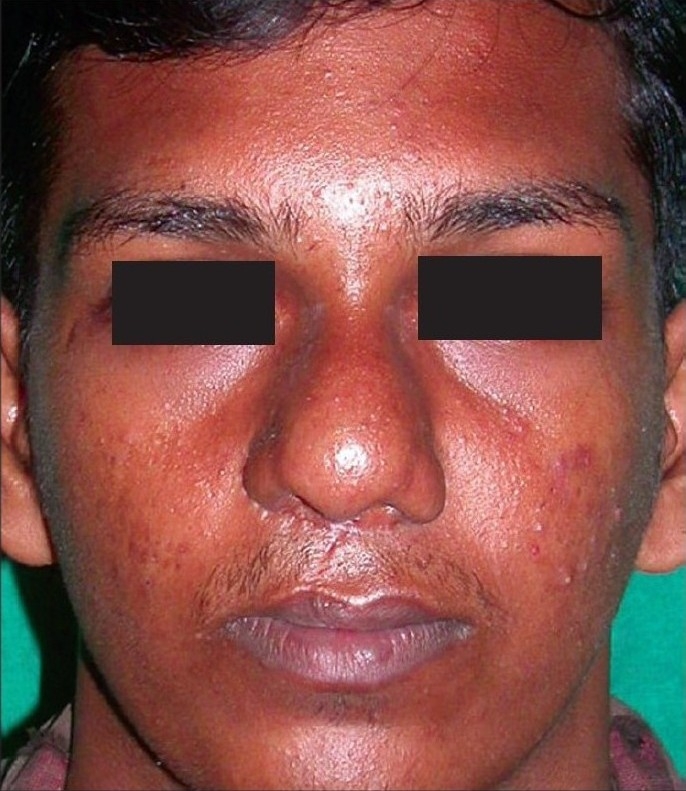
Post-op – long-term follow-up

### Primary closed rhinoplasty and extensive septal correction

Primary correction of the nasal deformity associated with the unilateral cleft lip has come to be accepted as the norm today. Many authors have recommended primary nasal correction[23–27] Some of them have used a closed approach.[23–25] Others have used an open technique.[24,26,27] There is also a group of authors who recommend a semi-open approach.[21] However, there is a consensus that some form of primary nasal correction must be done.

We use a closed rhinoplasty technique. With the help of Kilner's scissors, we approach the ala from both the medial and lateral aspects [Figure 10 a,b]. The medial approach is from the incision at the base of the columella. Laterally, the scissors are introduced at the base of the ala through the peri-alar incision. The scissors are used to dissect in the plane between the dorsal skin and the alar cartilages – both the lower and upper lateral cartilages are completely separated from the skin. The dissection is carried out till the nostril rim to free all superficial attachments of the alar cartilages. A more limited dissection is carried out on the non-cleft side up to the dome. The freed lower lateral cartilage is fixed to the upper lateral by means of bolster sutures.

**Figure 10a:**
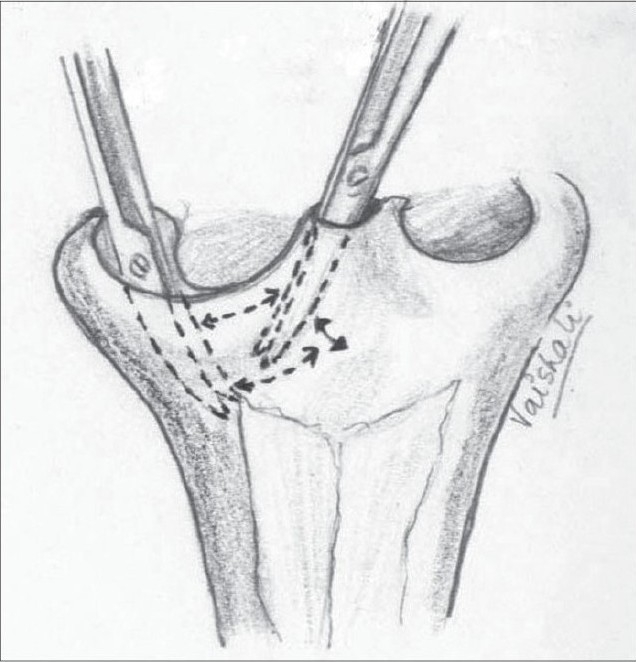
Closed alar dissection on the left side

**Figure 10b:**
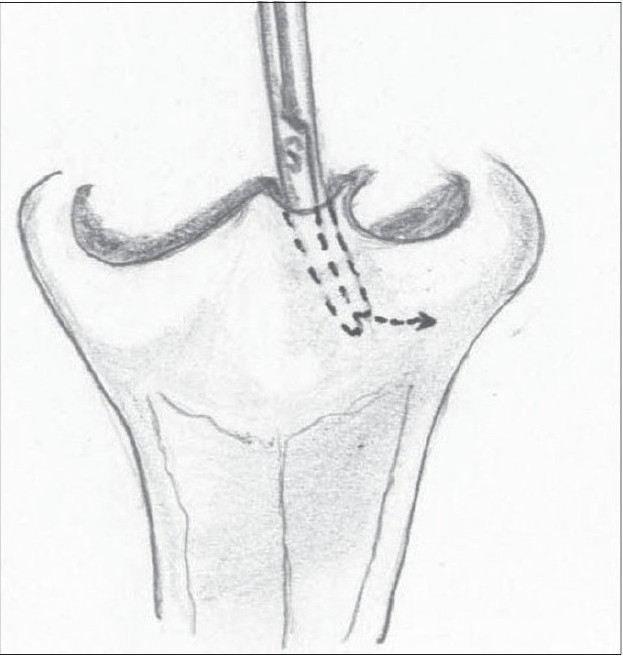
Closed alar dissection on the non-cleft side

**Figure 11:**
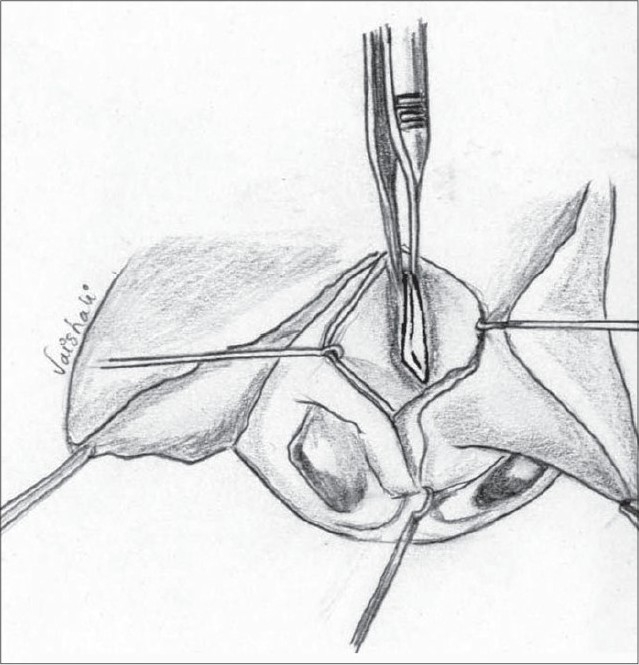
Septal cartilage dissected off the mucoperichondrium on both sides and from vomer and perpendicular plate of ethmoid

**Figure 12:**
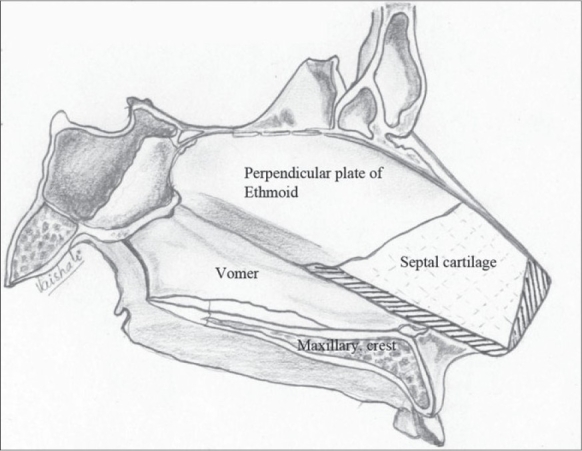
Septal cartilage showing the excised portion (shaded) and the scoring on the concave non-cleft side

**Figure 13:**
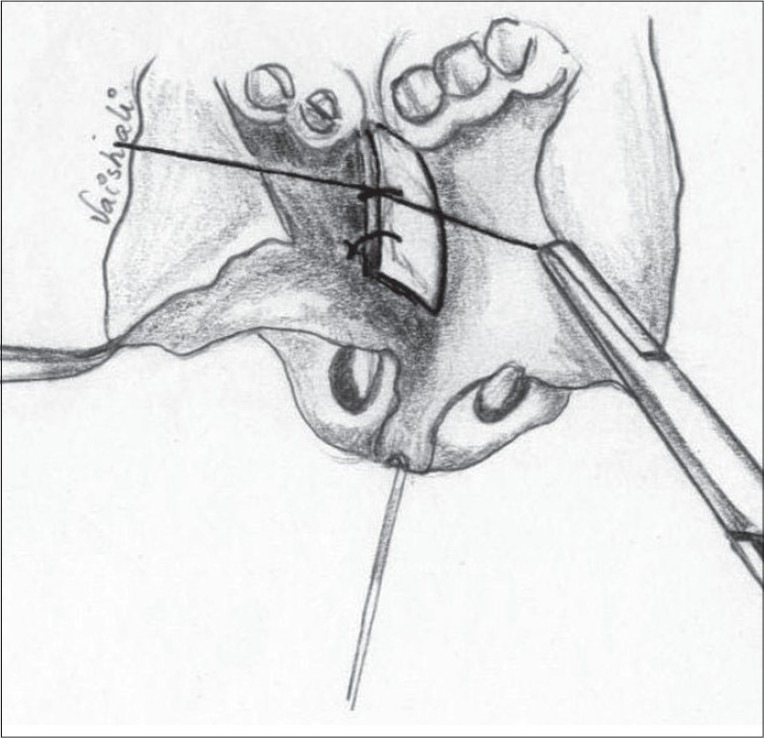
The septal cartilage is fixed to the nasal floor

The correction of the associated septal deviation is yet to gain universal acceptance. It is well documented that the septum is deviated towards the non-cleft side anteriorly.[28] The anterior nasal spine is itself similarly displaced to the non-cleft side. We approach the septum by incising over the mucoperichondrium on the cleft side on the groove at the base of the septum. The mucoperichondrium is carefully stripped off the septal cartilage. We then proceed to divide the septo-spinal ligament in order to expose the anterior border of the septal cartilage. This is an important step to avoid shearing of the septal cartilage when we proceed to strip it off the mucoperichondrium on the non-cleft side. This is done after incising the junction of the cartilages with the underlying maxillary crest. The septal cartilage is also freed from the vomer and the perpendicular plate of the ethmoid [Figure 11]. The cartilage thus freed will buckle when repositioned in the midline. Hence, a sliver of cartilage is excised inferiorly. We believe in Sir Harold Gillies' philosophy[29] that all cleft lip noses require some shortening. Hence we excise a thin wedge of septal cartilage anteriorly. This causes an upward recoil of the nasal tip, enhancing its projection. There is usually a residual bow-string effect to the cartilage even after all these manoeuvres. This is nullified by scoring with a knife on the non-cleft concave side of the cartilage until it is flail [Figure 12]. Finally, it is hitched to the newly reconstructed nasal floor on the cleft side to overcorrect the deviation, and with time it comes to lie in the midline [Figure 13]. A sliver of the excised cartilage is used as a vertical strut graft in the columella.

Many cleft surgeons have shied away from primary septal correction following apprehensions regarding the effect of this on subsequent nasal and maxillary development. However, the senior author has been following this radical septal correction for the past 40 years and we have not found any detrimental effect on any of our patients on long-term follow-up. In fact, we strongly believe that this has helped the overall functional outcome of the nose in our unilateral cleft lip patients. This has also been the view of other exponents like Samahel *et al*.[30] who have objectively studied the long-term effect using cephalograms. Other authors like Anderl[31] have confirmed that there is no additional deleterious effect in the long-term to maxillary or nasal growth from septal cartilage repositioning.

## OTHER REFINEMENTS

### High-riding nostril

Often, in patients who have a wide alveolar disparity between the medial and the lateral elements, we note that the nostril base on the cleft side comes to lie at a more superior level than its counterpart on the non-cleft side. This discrepancy has been corrected using an unequal Z plasty on the nasal floor as advocated by I.T. Jackson[32] [Figure 14 a,b]. Ever since we commenced using this additional procedure, the incidence of such high-riding nostrils has diminished dramatically. In children with severe alveolar disparities, sometimes we have had to perform two such Z plasties.

**Figure 14a:**
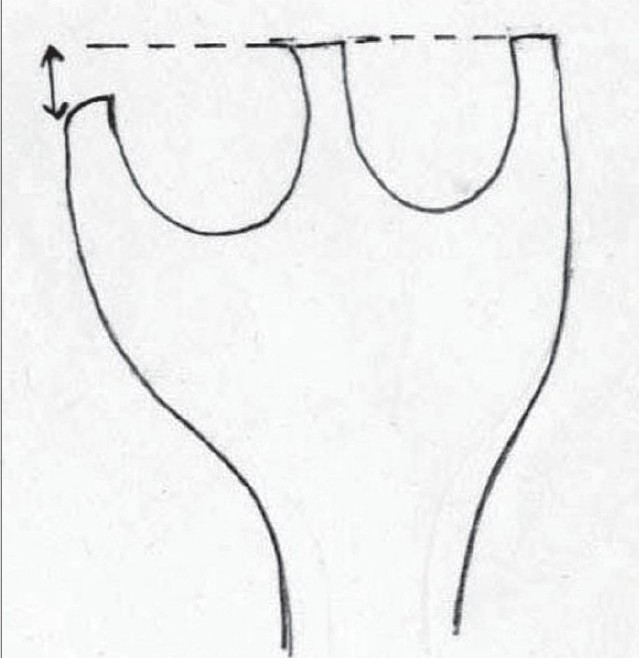
High-riding nostril

**Figure 14b:**
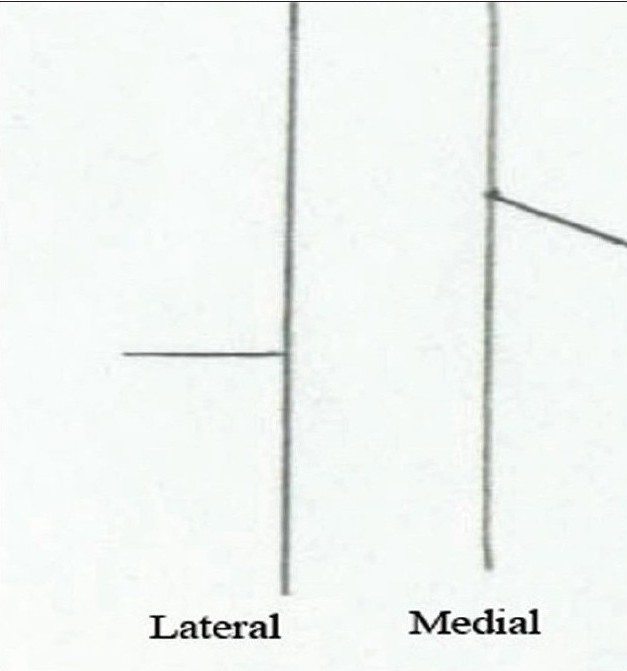
Jackson's Z plasty – lateral and medial

Another common deformity after a unilateral cleft lip repair is a deficiency in height of the lateral vermillion on the cleft side. This deformity has also been noted with other types of cleft lip repairs including the triangular repair. We believe that it is an inherent component of the unilateral cleft lip deformity and has nothing to do with the method of correction. As far as we know, there is no procedure documented so far to prevent this occurrence. However, it can secondarily be corrected either by a Gillies hemi-Cupid's bow procedure[14] or by a V-Y mucosal advancement.

### Unilateral partial cleft lip

The technique is essentially the same as for the complete variety. There is obviously no need for a nasal floor repair. However, there is some element of nasal deformity in most of these patients. Hence, we perform a closed alar cartilage dissection in all these patients. One needs to be wary while making the rotation and the back-cut as it is possible to lengthen the lip excessively.

### Microform cleft lip

These have variously been referred to as “a minimal cleft”,[33] “occult cleft”[34] “forme fruste cleft”[34] and “nature's union”.[35] The deformity always includes a vermillion notch. There may be, in addition, a white roll mal-alignment, a scar or a furrow on the body of the lip and a flattened alar cartilage with a wide nostril. When the deformity is confined to a notch of the vermillion, a notch correction procedure including muscle build-up and a Z plasty on the mucosa are all that is required. When there is no upward displacement of the Cupid's bow point, a simple straight repair (Rose-Thompson) would suffice.

However, in the more significant deformities that require downward rotation of the Cupid's bow point and closed nasal dissection, we follow Millard's procedure. But in the majority of these patients that have good muscle continuity across the cleft, we restrict the Millard incisions to the skin and subcutis and do not cut into the muscle. This form of a “cutaneous Millard's” rotation advancement procedure minimises the trauma inflicted on these patients with trivial deformities and helps in better scarring post-operatively. A muscle build-up is sometimes necessary. This innovation has been used by the senior author for many years now.

### Vestibular web

This is yet another vexing problem encountered during unilateral cleft lip repair. Some surgeons indulge in excision of the webbed vestibular skin and mucosa in the belief that there is an excess.[36] However, we believe that there is no real excess of vestibular lining. This is also the view held by other exponents.[14] The fold forms at the upper border of the lower lateral cartilage[14] and can only be eliminated if the lower lateral cartilage is hitched to the upper lateral. This may be done blindly in the closed technique or under vision in the open technique of primary nasal correction.

A Z plasty was described by Charles Pinto, mentor to the senior author[35], but remained unpublished till the present. [Figure 14 a,b]. The vertical limb of the Z is along the web. The two oblique (60°) limbs are then marked with the upper limb on the medial, and the lower limb on the lateral aspect. Care must be taken when elevating the vestibular lining flaps so that the underlying cartilage is not damaged. This procedure also helps in reorienting the axis of the nostril.

### Soft triangle deformity

With good primary nasal correction we have been able to consistently obtain acceptable results [Figures 3ab, 14ab,c 15ab]. However, in most of these cases, there remains a residual soft triangle droop. In many this is trivial. In some patients it is significant enough to require correction by a secondary rhinoplasty. With the present improved state of the art of secondary rhinoplasty, a good percentage of our patients are subjected to this procedure in an attempt to achieve well nigh perfection.

No cleft surgeon should forget the pathos of this deformity and the severe psychological trauma that it inflicts on parent and child. A plastic or reconstructive surgeon is really a general surgeon with a hobby and that hobby lies in the aesthetic realm of a refined reverence for tissue and the true appreciation of the dignity and beauty of the normal human form. His art would be quite meaningless if he reconstructed a face but failed to put a smile on it. The true plastic surgeon must always hope that the skill of his surgery will help towards the healing of all the internal scars that external wounds do cause.
